# Self-Catalyzed InSb/InAs Quantum Dot Nanowires

**DOI:** 10.3390/nano11010179

**Published:** 2021-01-13

**Authors:** Omer Arif, Valentina Zannier, Francesca Rossi, Daniele Ercolani, Fabio Beltram, Lucia Sorba

**Affiliations:** 1 NEST, Istituto Nanoscienze–CNR and Scuola Normale Superiore, Piazza San Silvestro 12, I-56127 Pisa, Italy; omer.arif@sns.it (O.A.); daniele.ercolani@sns.it (D.E.); fabio.beltram@sns.it (F.B.); lucia.sorba@nano.cnr.it (L.S.); 2 IMEM–CNR, Parco Area delle Scienze 37/A, I-43124 Parma, Italy; francesca.rossi@imem.cnr.it

**Keywords:** InSb quantum dots, nanowires, axial heterostructures, self-catalyzed growth

## Abstract

The nanowire platform offers great opportunities for improving the quality and range of applications of semiconductor quantum wells and dots. Here, we present the self-catalyzed growth of InAs/InSb/InAs axial heterostructured nanowires with a single defect-free InSb quantum dot, on Si substrates, by chemical beam epitaxy. A systematic variation of the growth parameters for the InAs top segment has been investigated and the resulting nanowire morphology analyzed. We found that the growth temperature strongly influences the axial and radial growth rates of the top InAs segment. As a consequence, we can reduce the InAs shell thickness around the InSb quantum dot by increasing the InAs growth temperature. Moreover, we observed that both axial and radial growth rates are enhanced by the As line pressure as long as the In droplet on the top of the nanowire is preserved. Finally, the time evolution of the diameter along the entire length of the nanowires allowed us to understand that there are two In diffusion paths contributing to the radial InAs growth and that the interplay of these two mechanisms together with the total length of the nanowires determine the final shape of the nanowires. This study provides insights in understanding the growth mechanisms of self-catalyzed InSb/InAs quantum dot nanowires, and our results can be extended also to the growth of other self-catalyzed heterostructured nanowires, providing useful guidelines for the realization of quantum structures with the desired morphology and properties.

## 1. Introduction

Semiconductor nanowires (NWs) provide an excellent platform for the exploitation of lattice-mismatched materials in high-quality heterostructures [1,2,3,4,5,6], since strain relaxation can occur efficiently along the NW sidewalls [7,8,9,10] and the critical thickness is several orders of magnitude higher than in conventional 2D growth. Consequently, NW-based heterostructures provide a wide range of opportunities for bandgap engineering combining different materials [1]. As an example, III–V semiconductor nanowires with embedded quantum dots (QDs) were realized and their integration in high-performance devices for electronics and optoelectronics applications has been demonstrated in the last few years [11,12,13,14,15,16,17]. The fabrication of QDs in NWs offers additional features over Stranski–Krastanow (SK) QDs, providing a tool for single QD control. This is a very important property that allows, for example, the implementation of a single-photon source for quantum cryptography and quantum computing [2,18,19]. Moreover, QDs in NWs are usually grown along the NW axis and this ensures the maximum collection efficiency and possibility of controlled coupling to a NW waveguide mode [2,19].

Among the various semiconductors, InSb is an attracting material for the fabrication of mid-infrared range optoelectronic devices (2–5 μm wavelength) [20], low-power high speed electronics [21], thermoelectric conversion [22], and quantum computation [20,23], due to its small bandgap, low effective masses, high electron mobility, and large thermo-power figure of merit [21,24,25,26]. InSb/InAs heterostructures represent one of the most promising material systems for optoelectronic devices operating in the mid-infrared range [27]. Moreover, InAs_x_Sb_1–x_ alloys provide the narrowest tunable bandgap for the infrared spectrum range, broadening the possible applications for devices operating in the mid and long wavelength infrared in emission or detection, especially for environmental gas detectors and security applications [25,28,29,30]. Since the last decade, much effort has been put into the growth of self-assembled InSb and InAsSb QDs [20,31,32,33,34,35,36]. However, the direct growth of InSb and InAsSb QDs of high crystal quality on commonly available semiconductor substrates is very challenging owing to the large lattice mismatch and to Sb segregation and surfactant effect [33,34]. Different approaches have been proposed for different types of substrate by introducing an intermediate layer of other materials in order to reduce the lattice mismatch [31,33] leading to very difficult growth protocols [20,31,33]. The NW geometry offers a great platform for improving the InAs/InSb material system quality and range of possible applications. However, the Au-assisted growth that is commonly employed for NW growth presents some limitations in the growth of double heterostructured nanowires, such as graded interfaces and kinking. In fact, it is challenging to preserve the stability of the catalyst nanoparticles (NPs) during material interchange [37,38,39]. In the specific case of InAs/InSb heterostructured NW, a straight InAs segment above the InSb segment is difficult to obtain, likely because of the change of composition of the Au–In alloy NPs [40]. The straight growth of the InAs axial segment was reported only above InAsSb segments up to intermediate antimony composition [40]. Furthermore, the Au-assisted growth method is not suitable for the growth of NWs on a complementary metal-oxide-semiconductor CMOS platform because Au is not compatible with CMOS-electronics. All these shortcomings can be overcome by embedding InSb QDs in catalyst-free or self-catalyzed InAs NWs. Here, we demonstrate for the first-time the successful growth of self-catalyzed InSb QDs embedded in InAs nanowires (refereed as InSb/InAs QD NWs hereafter) on Si (111) substrates. A systematic study on the influence of the growth parameters on the morphology of such NWs is conducted. Radial and axial growth rates are investigated as a function of growth parameters in order to obtain InSb QD with controlled morphology.

## 2. Methods

InSb/InAs QD NWs were grown on Si (111) substrates by means of chemical beam epitaxy (CBE) in a Riber Compact-21 system (Riber, Paris, France). The following metal-organic (MO) precursors were used for the growth: trimethylindium (TMIn), tert-butylarsine (TBAs), and tris-dimethyl-aminoantimony (TDMASb). The growth protocol consisted of three steps. In the first step, catalyst-free InAs NW stems were grown via the vapor-solid (VS) growth mechanism on Si (111). The complete description of substrate preparation and growth protocols is reported in references [41,42]. The growth of InAs stems was carried out for 30 min with the growth parameters previously optimized for the InAs/InSb axial NW heterostructures growth, i.e., line pressures of TBAs (*F_As_*) = 3.0 Torr and TMIn (*F_In_*) = 0.2 Torr, and growth temperature of 410 ± 5 °C. The average length (*L_stem_*) and edge-to-edge diameter (*D_stem_*) of the InAs NW stems were 360 ± 21 nm and 50 ± 4 nm, respectively. In the second step, In-assisted vapor-liquid-solid (VLS) InSb QDs were grown on top of these InAs NW stems [43] after a direct switch of the precursor fluxes, without any growth interruption. The InSb QDs were grown for 5 min with line pressures of TDMASb (*F_Sb_*) = 0.35 Torr and *F_In_* = 0.6 Torr at the same growth temperature of the InAs stems (410 ± 5 °C). The measured average length (*L_QD_*) and edge-to-edge diameter (*D_QD_*) of the InSb QDs were 40 ± 5 nm and 70 ± 4 nm, respectively. The contact angle *β* of the In NP on their tip was 118 ± 3° due to In-rich condition. Indeed, we chose these values of *F_In_* (0.60 Torr) and *F_Sb_* (0.35 Torr) based on our previous work on self-catalyzed InAs/InSb NWs [43] in order to provide stable conditions for the In droplet and the InSb QD shape and size, before the growth of the InAs top segment. In the third step, the growth of InAs top segments was performed: at the end of the InSb QD growth, both TMIn and TDMASb fluxes were simultaneously interrupted and the growth temperature for the InAs top segment was adjusted in 3 min. At this point, the growth of the InAs top segment was started by opening the TMIn and TBAs lines with previously adjusted line pressures. In order to study the morphology and to explore the growth mechanisms (VLS or VS) of the InAs top segments, three different series of samples were grown as a function of growth temperature, MO line pressures, and time duration. At the end of growth, the *F_In_* and *F_As_* fluxes were stopped and the sample was cooled down to 150 ± 5 °C in 3 min, in ultra-high vacuum (UHV) conditions (i.e., without TBAs flux). The NW morphology was characterized by scanning electron microscopy (SEM) in a Zeiss Merlin field emission microscope (Zeiss, Jena, Germany) operated at 5 keV. For imaging, the NWs were mechanically transferred from the as-grown substrates onto a Si substrate, in order to measure the different geometrical parameters from a 90° projection. Following this procedure, we could measure *D_QD_* and *L_QD_*, and these parameters at the end of the InAs top segment growth: the NW diameter specifically at the InSb QD position (*D_InSb_*), the base diameter of the NWs (*D_bottom_*), the total length of the NWs (*L_NWs_*), the base radius (*R*), and the height (*H*) of the nanoparticle (NP). All these quantities were averaged over ~25 NWs for each batch. From these measured parameters we could calculate the NP volume (*V_NP_*) and contact angle (*β*) [44,45], the InAs shell thickness around the InSb QD (*t_shell_* = (*D_InSb_* − *D_QD_*)/2), and the length of InAs top segment (*L_top_* = *L_NWs_* − (*L_stem_* + *L_QD_*)). The crystal structure of the grown NWs was characterized by transmission electron microscopy (TEM) using a JEOL JEM-2200FS microscope (JEOL, Tokyo, Japan) operated at 200 keV, equipped with an in-column Ω filter. Imaging was performed in scanning TEM (STEM) mode and in high-resolution (HR) TEM mode combined with zero-loss energy filtering. For TEM characterization, the NWs were mechanically transferred to carbon-coated copper grids.

## 3. Results and Discussion

### 3.1. InAs Growth Temperature Series

**Figure 1:**
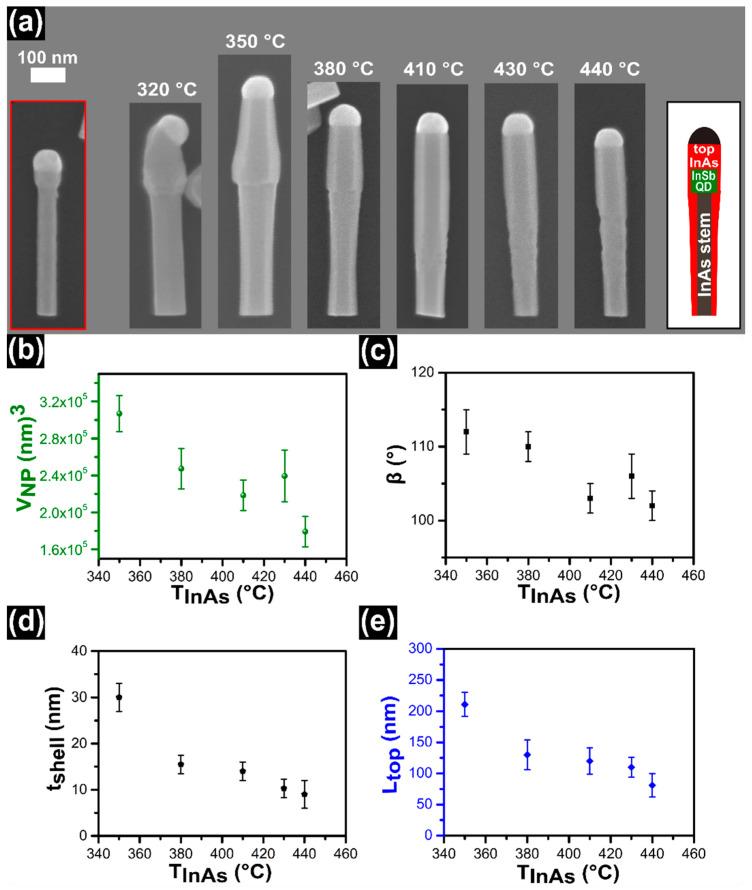
(**a**) Series of scanning electron microscope (SEM) images of the InSb/InAs quantum dot (QD) nanowires (NWs). The first NW shown in the red frame represents the InAs/InSb NW grown at 410 ± 5 °C. The other panels represent the InSb/InAs QD NWs where the InAs top segment has been grown with the line pressures *F_In_* = 0.30 Torr and *F_As_* = 0.25 Torr and growth time 15 min at different growth temperatures (*T_InAs_*) as indicated in each panel. The drawing in the right side of the panel shows a schematic picture of the InSb/InAs QD NW structure. (**b**) Evolution of the NP volume as a function of *T_InAs_*. (**c**) Evolution of contact angle (*β*) of the In NPs on the top of NWs as a function of *T_InAs_*. (**d**) Evolution of the InAs shell thickness as a function of *T_InAs_* which represents the InAs radial growth around the InSb QD. (**e**) Evolution of length of InAs top segments (*L_top_*) as a function of *T_InAs_*. The symbols represent an average value of each sample obtained by measuring ~25 NWs and error bars represent the standard deviation of the average.

Initially, we studied the influence of the growth temperature (*T_InAs_*) on the evolution of InAs top segment. In this set of experiments, the line pressures of 0.3 Torr and 0.25 Torr for *F_In_* and *F_As_* respectively, and the growth time of 15 min were kept constant, while the growth temperature of top InAs was varied (*T_InAs_* = 320, 350, 380, 410, 430, and 440 ± 5 °C). SEM images of one representative InSb/InAs QD NW from each sample are presented in Figure 1a. In the first red framed box we show the InAs/InSb NW before the growth of the InAs top segment. We can clearly see the In droplet on the top of NW, the InSb QD with larger diameter and the InAs stem with uniform diameter. The InSb QD shows the same kind of side facets of the InAs stem (i.e., {110} [8,46]). The other NWs depicted in panel (a) are representative SEM images of InSb/InAs QD NWs where the InAs top segment was grown at different temperatures, *T_InAs_*, as indicated in the figure. The presence of In NPs on the top of all NWs reveals a wide range of temperature window for the In-assisted InAs VLS growth. The InAs top segment grows with the same kind of side facets of InSb QD and InAs stem. In all samples, the NWs show the growth axis aligned with the <111> crystallographic direction and a straight, perpendicular NW/NP interface, except the sample grown at *T_InAs_* = 320 ± 5 °C in which all NWs exhibit tilted NW/NP interface. This can be ascribed to the development of a more stable growth interface at this temperature, or to the instability of the droplet triggered by its larger volume [47,48].

We analyzed the In particle on the top of the NWs and we calculated its volume by considering it as a spherical cap having contact angle *β* and a base radius *R*. The NP volume is given by *V_NP_* = (*πR*^3^/3) *f*(*β*), with *f*(*β*) = [(1 − cos *β*) (2 + cos *β*)]/[(1 + cos *β*) sin *β*] [43]. The evolution of volume and *β* of the In NP as a function of *T_InAs_* are plotted in Figure 1b,c, respectively. It is observed from the plots that both volume and contact angle of In NP decrease with increasing growth temperature of InAs top segment.

Figure 1d shows instead the relationship between the InAs shell thickness around the QD (*t_shell_*) and *T_InAs_*. Notably, the shell thickness decreases by increasing the growth temperature from 350 to 440 ± 5 °C. The minimum value of shell thickness of 9 ± 3 nm is achieved at 440 ± 5 °C. Finally, the dependence of the InAs top-segment length (*L_top_*) on *T_InAs_* is shown in Figure 1e. The longest InAs top segment with *L_top_* (210 ± 19 nm) is obtained at *T_InAs_* = 350 ± 5 °C then it decreases. The minimum value of *L_top_* (81 ± 19 nm) is measured at *T_InAs_* = 440 ± 5 °C. All the other measured parameters, as *D_InSb_*, *D_bottom_*, *L_NWs_*, as a function *T_InAs_* are described in Figure S1 of the supporting information (SI) and they confirm that both axial and radial growth (around the InSb dot and the InAs stem) during the InAs top segment deposition decrease by increasing the growth temperature. It should be mentioned that in most of the samples, the position of the InSb QD was easily localized by SEM images, thanks to its wider diameter. In the samples in which the InAs/InSb interface was not clearly visible, we used energy-dispersive X-ray spectroscopy (EDX) at SEM to localize the InSb QD (following the drop of As signal in the elemental line profile) and then we measured the NW diameter at that position by SEM imaging.

### 3.2. As Line Pressure Series

**Figure 2:**
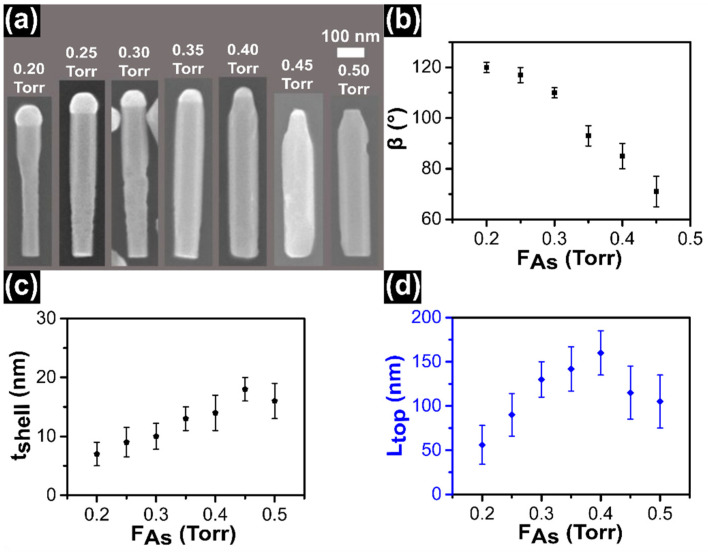
(**a**) SEM images of InSb/InAs QD NWs obtained with fixed line pressures *F_In_* = 0.30 Torr, growth time 15 min and *T_InAs_* = 440 ± 5 °C, while *F_As_* varied from 0.20 Torr to 0.50 Torr as indicated in each panel. The droplet volume decreases with increasing *F_As_* and finally there is no droplet in the sample grown with *F_As_* = 0.50 Torr. (**b**) Plot of contact angle (*β*) of In droplets versus *F_As_*. (**c**) Evolution of shell thickness around the InSb QD versus *F_As_*. (**d**) Evolution of length of top InAs segments (*L_top_*) as a function of *F_As_*. The symbols represent an average value of each sample obtained by measuring ~25 NWs and error bars represent the standard deviation of the average.

In the second set of experiments, we selected *T_InAs_* = 440 ± 5 °C, which was found to minimize the shell thickness around the InSb QD and we varied *F_As_* from 0.20 Torr to 0.50 Torr. The growth time and *F_In_* were set to 15 min and 0.30 Torr, respectively. Figure 2a shows SEM images of representative NWs of this series of samples. First we observed that the NP volume decreases with increasing *F_As_* and the NP base radius shrinks above 0.35 Torr, yielding a pencil like shape of the NW tips. Then we noticed that the length of InAs segment increases with increasing *F_As_* up to 0.40 Torr. For *F_As_* > 0.40 Torr the length starts to decrease, moreover at 0.50 Torr there is no particle on the top of the NWs. It is well known in self-catalyzed growth that the group III particle starts to shrink with increasing groupV flux and can be completely consumed in group V-rich condition. The contact angle of the In droplet on the top of NWs as a function of *F_As_* is plotted in Figure 2b. The contact angle slowly decreases with increasing *F_As_* line pressure. It remains greater than 90° for *F_As_* < 0.35 Torr, while for higher *F_As_* it becomes smaller than 90°. The evolution of contact angle confirms the consumption of In NP with increasing *F_As_*. We calculated the effect of *F_As_* on the evolution of the NW shape, i.e., on the radial and axial InAs growth. Both NW diameter around the QD and the base diameter increase with increasing *F_As_* but with different slopes (see Figure S2a of SI) and this explains why for *F_As_* 0.50 Torr the NW diameter is uniform along the whole length.

We plotted the InAs shell thickness around InSb QD as a function of *F_As_* in Figure 2c. It can be seen that the shell thickness increases with increasing *F_As_*. This suggests that the radial growth of InAs around the InSb QD is As-limited and is directly proportional to *F_As_*. Another possible explanation is that the high As line pressure reduces the desorption rate of In adatoms [49,50]. Hence, the sticking probability of In adatoms on the NW sidewalls is enhanced at high *F_As_*, which increases the radial growth rate of InAs. These findings are consistent with previously reported results in the case of self-catalyzed InAs [51] and GaAs NWs [52,53].

The influence of As line pressure on *L_top_* is plotted in Figure 2d, while *L_NWs_* is shown in the SI (Figure S2b). For the length of InAs top segment, two distinct regions are observed. The axial growth rate increases with increasing As line pressure up to 0.40 Torr. Here it shows a maximum value of 160 ± 32 nm, and above 0.40 Torr *L_top_* decreases. The decrement of *L_top_* is ascribed to the consumption of the In NP on the top of NWs. Indeed, the axial-growth mechanism of the NWs is VLS for As line pressure from 0.20 Torr to 0.4 Torr, and the axial growth rate is known to be directly proportional to the As flux in self-catalyzed InAs NWs [54]. At *F_As_* = 0.45 Torr the In particle is still visible (still VLS growth mode), but its volume and contact angle are strongly reduced, explaining why the axial growth rate decreases. Finally, for As line pressure higher than 0.45 Torr the growth mode becomes VS due to the absence of NP on the top of NWs and the axial growth rate drops.

### 3.3. Time Series

**Figure 3:**
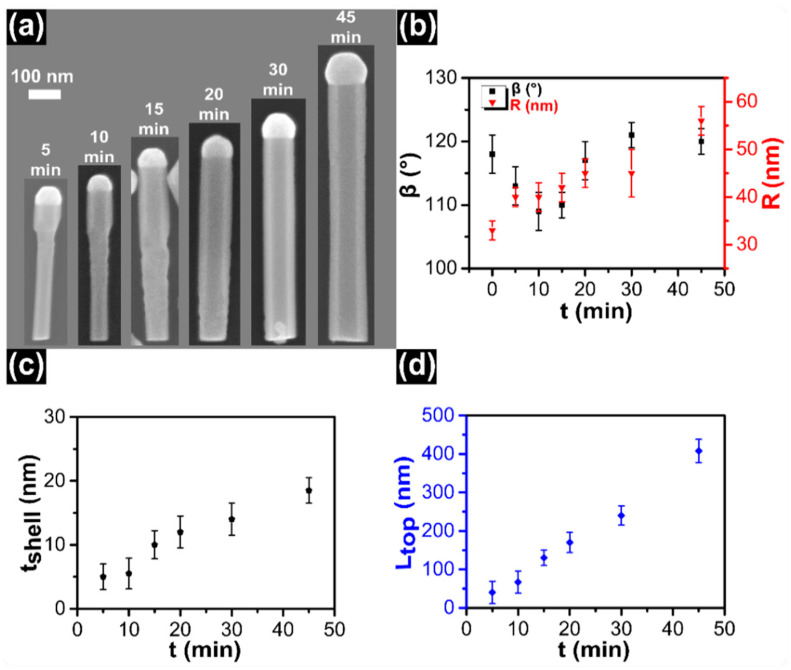
(**a**) SEM images of representative InSb/InAs QD NWs, obtained with fixed line pressures *F_In_* = 0.30 Torr, *F_As_* = 0.30 Torr, at *T_InAs_* = 440 ± 5 °C, while the growth time of InAs top segments varies from 5 min to 45 min as indicated in each panel. (**b**) The evolution of contact angle of In droplets on the top of NWs and the base radius of NP as a function of growth time. At time *t* = 0 min they correspond to contact angle and base radius at the end of InSb segment growth. (**c**) Evolution of InAs shell thickness around the InSb QD as a function of growth time. (**d**) Evolution of length of InAs top segments as a function of growth time. The symbols represent an average value of each sample obtained by measuring ~25 NWs and error bars represent the standard deviation of the average.

In the last set of experiments, the time evolution of InAs top segment under In-rich conditions was studied. In this series of samples, the line pressures of *F_In_*, *F_As_*, and growth temperature were kept constant at 0.30 Torr, 0.30 Torr, and 440 ± 5 °C, respectively, while the growth time of InAs top segments was varied as *t* = 5, 10, 15, 20, 30, and 45 min. The representative SEM images from each sample are shown in Figure 3a. An In droplet is found on the top of all NWs: this is evidence of self-catalyzed VLS growth mechanism persisting at all growth times. As clearly seen from the SEM images, the InAs top segment elongates axially and, at the same time, there is a shell growth along the whole NW length that occurs with different rates around the InSb QD and around the InAs stem. Indeed, after 20 min of growth time, the InAs/InSb interface is not visible any more because the NW diameter is uniform from the bottom to the top.

The evolution of the contact angle and base radius of the NP as a function of growth time are plotted in Figure 3b. At zero time (representing the InAs/InSb NW without any InAs top segment), the calculated values of the contact angle and the base radius are 118 ± 3° and 33 ± 2 nm, respectively. At the beginning of InAs top segment growth, for 0 < *t* < 15 min, the contact angle decreases. This behavior is probably associated with an increment of the base radius of the NP/NW interface that is faster than the increase of NP size at the early growth stages [43,55,56]. Then, the contact angle slightly increases with growth time and it stabilizes around 120 ± 3°. We measured also *D_InSb_*, *D_bottom_*, and *L_NWs_* as a function of the InAs top segment growth time. The corresponding values are plotted in Figure S3 of SI.

The calculated InAs shell thickness around the InSb QD as a function of growth time is shown in Figure 3c. The shell thickness increases linearly with increasing growth time. The growth rate of the InAs shell around the InSb QD can be derived from the slope of the linear fit of experimental data points. We obtain a radial growth rate of 0.46 ± 0.04 nm/min. The influence of growth time on the length of InAs top segments is plotted in Figure 3d. Also in this case we observe that the length of InAs top segments increases linearly with increasing growth time. We have not observed any delay or incubation time and saturation of NW length up to 45 min of growth time. The absence of incubation time is attributed to the In droplet already formed on top of the InSb segment. The axial growth rate can be obtained from the slope of the linear fit of experimental data points whose intercept is passing through the origin. We obtain an axial growth rate of 8.60 ± 0.25 nm/min. The axial growth rate is much higher as compared to the radial growth rate, as typically observed in VLS versus VS growth. However, the length elongation is always accompanied by an expansion in diameter as commonly observed in self-catalyzed NW growth. 

### 3.4. Nanoparticle Consumption

**Figure 4:**
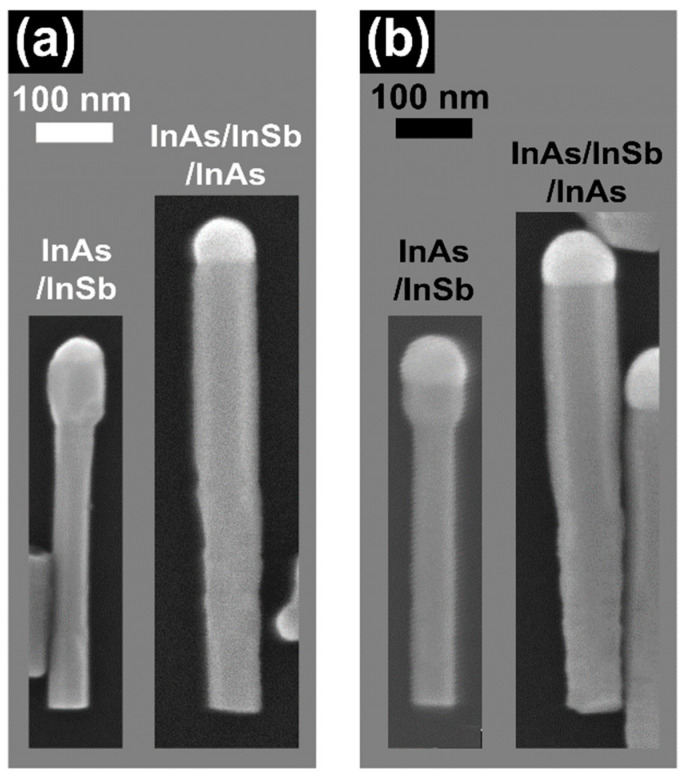
(**a**) SEM images of InAs/InSb heterostructured NW (left) and InSb/InAs QD NWs (right), obtained with partial consumption of In NP under *F_As_* = 1 Torr for 1 min. (**b**) SEM images of InAs/InSb heterostructured NW (left) and InSb/InAs QD NWs (right) obtained without consumption of the In NP. In both cases the growth of the top InAs segment was performed with line pressures of *F_In_* = 0.30 Torr *F_As_* = 0.30 Torr, and growth time of 15 min at *T_InAs_* = 440 °C.

In order to better understand the InAs radial growth mechanisms and explain why the growth rate is different around the InAs stem and the InSb QD, we conducted an additional experiment. The idea of this experiment was to partially consume the In NP on top of the InAs/InSb NWs in order to start the InAs top segment growth with a smaller NP. In this experiment, the InAs/InSb NWs were kept under the As line pressure of 1 Torr for 1 min at 410 ± 5 °C at the end of the InSb QD growth, to partially consume the In NP. Indeed, this procedure reduces the NP size and the contact angle *β,* that previously was around 120°, becomes smaller than 90°. Subsequently, the growth of InAs top segment was carried out using the same growth condition: *F_In_* = 0.30 Torr and *F_As_* = 0.30 Torr for 15 min at *T_InAs_* = 440 ± 5 °C. Figure 4 shows the SEM images of nanowires of the samples with (a) and without (b) consumption of the In NP, before (left) and after (right) the InAs top segment growth. All the measured parameters are listed in Table 1.

By comparing the values of *D_InSb_* in the two samples with the InAs top segment, we can clearly see that *D_InSb_* is smaller (79 ± 6 nm) in the sample grown after In NP consumption, compared to the sample grown without NP consumption (90 ± 4 nm), while the value of *D_bottom_* is the same in both samples (66 ± 6 and 68 ± 5 nm). Therefore, we can conclude that the In-droplet size plays a role in the InAs radial growth around the InSb QD, while it does not affect the InAs radial growth at the NW bottom. This suggests that there are different In adatom diffusion paths towards the growth front: one from the substrate, that is independent from the NP size, and one from the NW sidewalls, involving only a collection area close to the NP (within a diffusion length from the droplet), and directly influenced by the NP dynamics [57].

### 3.5. Discussion

**Figure 5:**
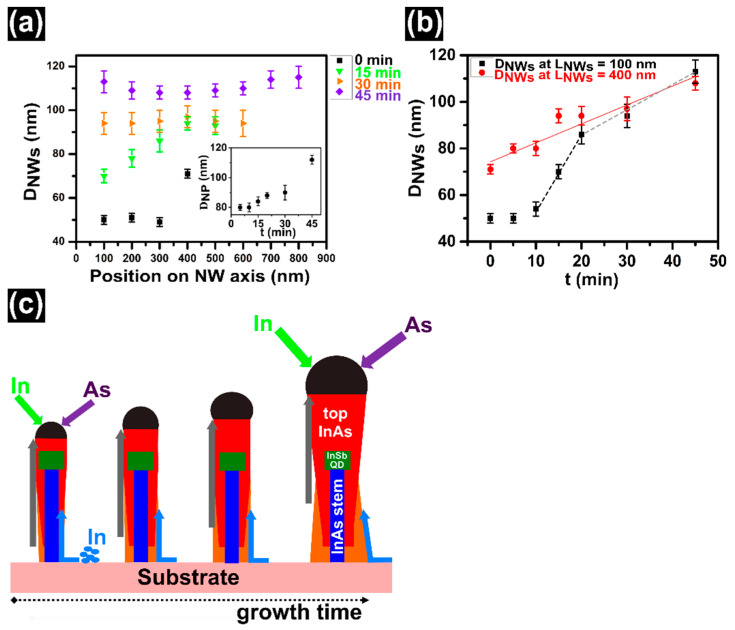
(**a**) NW diameter as a function of position along the length of NW, for 0, 15, 30 and 45 min samples of the time series shown in Figure 3a. The inset of panel (**a**) shows the relationship between *D_NP_* and growth time. (**b**) Evolution of the diameter of NWs at the base of NWs (*L_NWs_* 100 ± 4 nm) and around the InSb QD (*L_NWs_* 400 ± 4 nm) with respect to the growth time. The red line represents the linear fit of the *D_InSb_* versus time, while the black and grey dotted lines are just to guide the eye and highlight the change of slope. (**c**) A schematic illustration of the growth mechanisms for the radial growth (the blue and gray arrows indicate the two In adatom diffusion paths, from the substrate and from the NW sidewall within a diffusion length from the NP, respectively) and the resulting shape of the NWs versus growth time.

The role of the In NP is even more evident if we consider the diameter evolution of the NWs at different positions along the growth axis. Indeed, we measured the diameter of the NWs from the base to the tip, every 100 ± 4 nm, along the growth axis, for all samples of the time series shown in Figure 3. In particular, the results for 0, 15, 30, and 45 min samples are plotted in Figure 5a. Considering the lower part of the NW (below the QD position, i.e., below 400 nm), it is clear that it has a uniform diameter at the beginning of the InAs top segment growth, then it starts to be inverse-tapered at 15 min of growth and continues to be inverse tapered below 30 min. The diameter then becomes uniform for 30 min growth and it evolves as tapered at 45 min of growth. On the other hand, the diameter of the upper part of the NW (above the QD position) is uniform up to 30 min of InAs top segment growth and it becomes inverse-tapered at 45 min of growth.

Figure 5b shows the NW diameter versus InAs growth time for 2 positions along the growth axis: at 100 ± 4 nm and at 400 ± 4 nm from the base. The former representing the diameter of the lower part of the InAs stem and the latter the diameter of the NW at the InSb QD position, i.e., *D_InSb_*. We found that *D_InSb_* increases almost linearly with growth time, with constant slope, suggesting a constant growth mechanism, that is probably the diffusion of the In adatoms on the NW sidewalls close to the NP (within a diffusion length from the droplet). Conversely, the diameter of the NWs at 100 nm from the base, after an initial delay, increases quickly from 10 min to 20 min of growth time and then it continues to increase but with an abrupt change of slope. This behavior suggests that for the first 20 min of growth there are two contributions to the InAs radial growth in the lower part of the NWs, i.e., the diffusion of the In adatoms from the substrate and the diffusion on the NW sidewalls toward the growth front. Above 20 min of growth (*L_NWs_* > 570 nm) the In droplet is too far from the NW base so that sidewall diffusion within the collection area close to the NP does not contribute anymore to the radial growth at the NW base. Indeed, above 30 min of InAs growth the only contribution to the radial growth at the lower part of the NWs is the diffusion from the substrate and we observe a gradual tapering. This suggests a diffusion length of the In adatoms lower than 280 nm, consistent with the tapering shape observed at the NW lower segment in the 45 min sample. A schematic illustration of the combination of the two mechanisms for the InAs radial growth and its effect on the final NW shape is shown in Figure 5c. Similar results were reported for Ga-assisted GaAs [58] and GaP [59] NWs.

Now, considering all these results, we can provide a general description of the axial and radial growth of the InAs top segment: the *T_InAs_* and *F_As_* series, and the NP-consumption experiment clearly demonstrate that the volume of the In NP is playing an important role for both the axial and the radial growth of the InAs top segment. Indeed, at low growth temperature the NP volume is larger, therefore the radial growth in the top part of the NW is enhanced [57]. At the same time, the VLS axial growth is also enhanced by bigger NPs. As the growth temperature is increased, the NP become smaller and so both axial and radial growth rate decrease. Moreover, at high growth temperature In desorption starts to be sizable [60] so that both growth rates decrease. Moreover, considering the As-line pressure series, we found that when the growth conditions preserve an In droplet with *β >* 90° on top of the NWs, both axial and radial growths are enhanced by increasing *F_As_*, meaning that both VLS and VS growth are As-limited. Finally, the time evolution of the NW diameter for samples growth under In-rich conditions allowed us to confirm that there are two contributions to the radial InAs growth, i.e., diffusion of In adatoms from the substrate toward the growth front, and the diffusion on the NW sidewalls within a diffusion length distance from the NP. The second contribution is strongly affected by the NP size and dynamics, as it is known to be for self-catalyzed NWs [57]. The two diffusion paths and the total length of the NW determine the final shape of the NW.

### 3.6. Study of Crystal Structure

**Figure 6:**
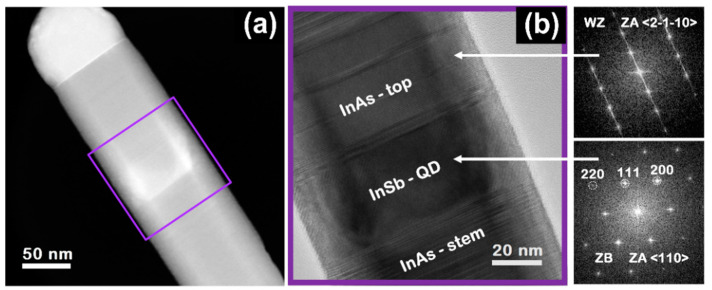
TEM analysis of a representative InSb/InAs QD NW. (**a**) high-angle annular dark field scanning transmission electron microscopy (HAADF-STEM) image, (**b**) high-resolution TEM (HR-TEM) image of the NW region framed by the purple square in panel (**a**). The fast Fourier transform (FFT) analysis shown on the bottom right confirms the ZB structure of the QD segment.

In order to study the crystal structure of the InSb/InAs QD NWs, we performed TEM analysis. Figure 6 shows the high-angle annular dark field (HAADF)-STEM (a) and HR-TEM with the corresponding fast Fourier transforms (FFT) (b) images of the InSb/InAs QD NWs with the InAs top segment grown with *F_In_* = 0.30 Torr, *F_As_* = 0.25 Torr, for *t* = 15 min at the growth temperature of 440 °C. From the STEM micrograph we could identify the InSb QD thanks to the different Z contrast of InAs and InSb materials and we could measure the diameter and length of InSb QD and length of InAs top segment obtaining *D_InSb_* = 85 ± 4 nm, *L_QD_* = 39 ± 6 nm, and *L_top_* = 105 ± 5 nm, respectively, consistent with the results obtained from SEM images. From the HR-TEM analysis, we observed that InAs-stem and InAs-top segments have a wurtzite (WZ) crystal structure with several defects such as stacking faults and twins perpendicular to the growth direction. It is commonly observed that the InAs NWs grown by catalyst-free and self-catalyzed growth methods show highly defective (or mixed wurtzite/zinc blende) crystal structure [41,54]. By contrast, the InSb QD shows a defect-free zinc blende (ZB) crystal structure without any stacking fault, consistently with the energetically preferred cubic structure of the InSb crystals [23,25,29,43] generally attributed to the low ionicity of group III to Sb bonds [25]. This analysis confirms the good crystal quality of the self-catalyzed InSb QDs embedded in InAs NWs.

## 4. Conclusions

In conclusion, we have demonstrated the growth of self-catalyzed InSb/InAs QD NWs on Si (111) substrates by a chemical beam epitaxy technique. The morphology and the growth mechanisms of the InAs top segment were thoroughly investigated as a function of growth temperature, As line pressure, and growth time. We found that both axial and radial growth rates decrease by increasing the growth temperature. Furthermore, both axial and radial growth rates are enhanced by the As line pressure, therefore both the VLS axial growth and the VS radial growth are As-limited in a self-catalyzed growth regime. Finally, we found that the volume of the In NP is strongly affecting both the axial and the radial growth of the InAs top segment. Indeed, the radial growth around the InSb QD and the top part of the NW is mainly determined by the volume of the In NP, while the radial InAs growth at the bottom part of the NWs is affected from both the NP size and the surface diffusion of In adatoms from the substrate below a certain NW length, then becoming independent from NP dynamics. Our study provides useful guidelines for the realization of InSb/InAs QD NWs with the desired shape and morphology for device applications. Moreover, we believe that the results obtained could be useful for engineering the properties of other self-catalyzed NWs systems.

## Figures and Tables

**Table 1:** Measured and calculated geometrical parameters for the samples reported in Figure 4.

Type of Growth	Growth Conditions	*R* (nm)	*H* (nm)	*β* (°)	*D_Insb_* (nm)	*D_bottom_* (nm)	*L_NWs_* (nm)
InAs/InSb	Without In NP consumption	33 ± 2	54 ± 4	118 ± 3	70 ± 4	50 ± 4	400 ± 21
InAs/InSb	With In NP consumption	32 ± 2	31 ± 3	88 ± 5	75 ± 3	51 ± 3	460 ± 35
InAs/InSb/InAs	Without In NP consumption	44 ± 2	67 ± 5	113 ± 3	90 ± 4	68 ± 5	535 ± 33
InAs/InSb/InAs	With In NP consumption	36 ± 4	50 ± 7	109 ± 3	79 ± 6	66 ± 6	555 ± 40

## Data Availability

Data is contained within the article or supplementary material

## Supplementary Materials

The following are available online at https://www.mdpi.com/2079-4991/11/1/179/s1, Figure S1: InAs temperature series, Figure S2: *F_As_* series, Figure S3: Time series.
