# Supplementary material for: Self-Catalyzed InSb/InAs Quantum Dot Nanowires
Source: Nanomaterials (Basel). 2021 Jan 13;11(1):179. doi: 10.3390/nano11010179 (PMC7828319; doi:10.3390/nano11010179)
Supplement: Supplementary file 1 [file nanomaterials-11-00179-s001.pdf]

nanomaterials

Supplementary material

Self-Catalyzed InSb/InAs Quantum Dot Nanowires

Omer Arif [1], Valentina Zannier [1,*], Francesca Rossi [2], Daniele Ercolani [1], Fabio Beltram [1] and Lucia Sorba [1]

[1] NEST, Istituto Nanoscienze—CNR and Scuola Normale Superiore, Piazza San Silvestro 12, I-56127 Pisa, Italy; omer.arif@sns.it (O.A.); daniele.ercolani@sns.it (D.E.); fabio.beltram@sns.it (F.B.); lucia.sorba@nano.cnr.it (L.S.)
[2] IMEM—CNR, Parco Area delle Scienze 37/A, I-43124 Parma, Italy; francesca.rossi@imem.cnr.it
* Correspondence: valentina.zannier@nano.cnr.it; Tel.: +39-050-509-123(474)

In order to measure the geometrical parameters, the nanowires (NWs) were mechanically transferred from the as-grown substrates onto a Si substrate and 90° projection SEM images were performed. We measured the following quantities for each sample (averaged over ~ 25 NWs): the length (L_{QD}) and the diameter (D_{QD}) of the InSb QD, the diameter of the NWs specifically around InSb QD (D_{InSb}), the NW total length (L_{NWs}), and its base diameter (D_{bottom}), the base radius (R), the height (H), and contact angle (β) of the nanoparticle (NP) on top of the NWs. In the case of InAs/InSb NWs (without InAs top segment), the measured values of D_{InSb}, D_{bottom}, and L_{NWs} were 70 ± 4 nm, 50 ± 4 nm, and 400 ± 21 nm, respectively. These values are applicable for all the samples of the series.

S1. InAs Temperature Series

In Figure S1, we show the plots of D_{InSb}, D_{bottom} and L_{NWs} as a function of T_{InAs} for the series of the sample in which we varied only the growth temperature of InAs top segment (Figure 1 of the main text).

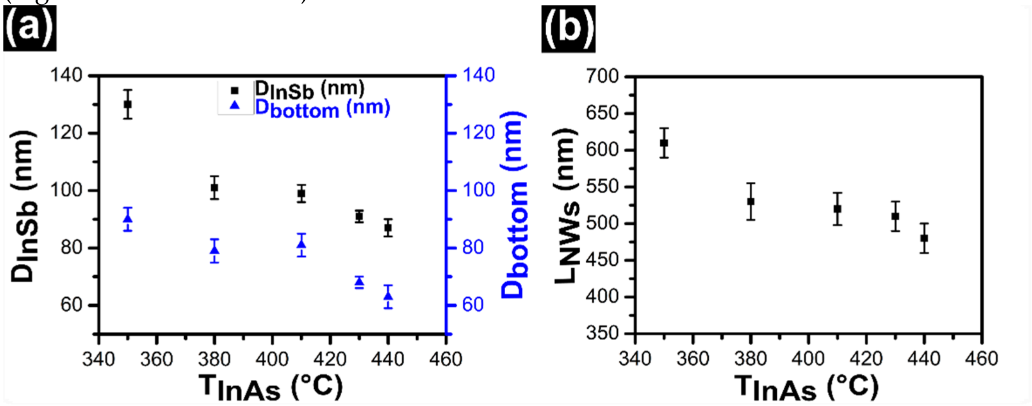

Figure S1. (**a**) Evolution of the diameter of the NW around the InSb QD and at the bottom versus T_{InAs}. (**b**) Evolution of total length of NWs (L_{NWs}) as a function of T_{InAs}. The symbols represent an average value of each sample obtained by measuring ~ 25 NWs and error bars represent the standard deviation of the average. .

S2. As Line Pressure Series

In Figure S2, we show the plots of D_{InSb}, D_{bottom} and L_{NWs} as a function of As line pressure F_{As} for the series of the sample in which we varied only the As line pressure during the growth of InAs top segment (Figure 2 of the main text).

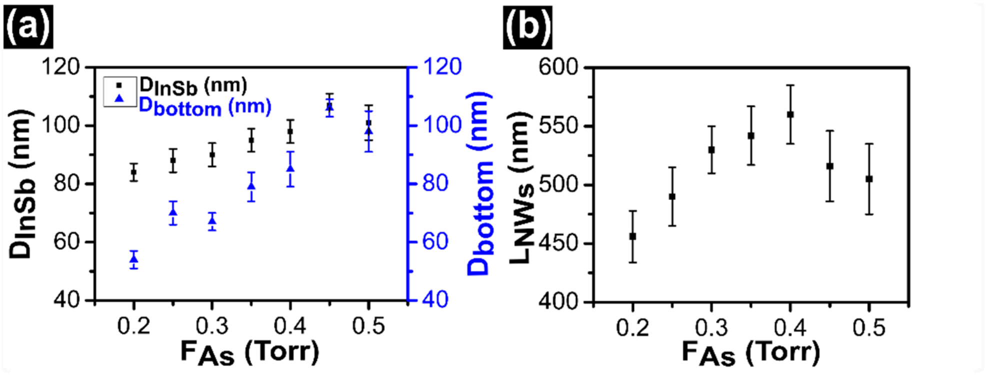

Figure S2. Morphological parameters of InSb/InAs QD NWs, obtained with fixed In line pressure F_{In} = 0.30 Torr, growth time 15 min at T_{InAs} = 440 °C, while F_{As} varies from 0.20 Torr to 0.50 Torr, (**a**) Expansion of NW diameter at the InSb QD position and at the bottom of NWs versus F_{As}. (**b**) Evolution of total length of NWs (L_{NWs}) as a function of F_{As}. The symbols represent an average value of each sample obtained by measuring ~ 25 NWs and error bars represent the standard deviation of the average.

S3. Time Series

In Figure S3, we show the plots of D_{InSb}, D_{bottom}, and L_{NWs} as a function of InAs growth time for the series of the sample in which we varied only the growth time of InAs top segment (Figure 3 of the main text).

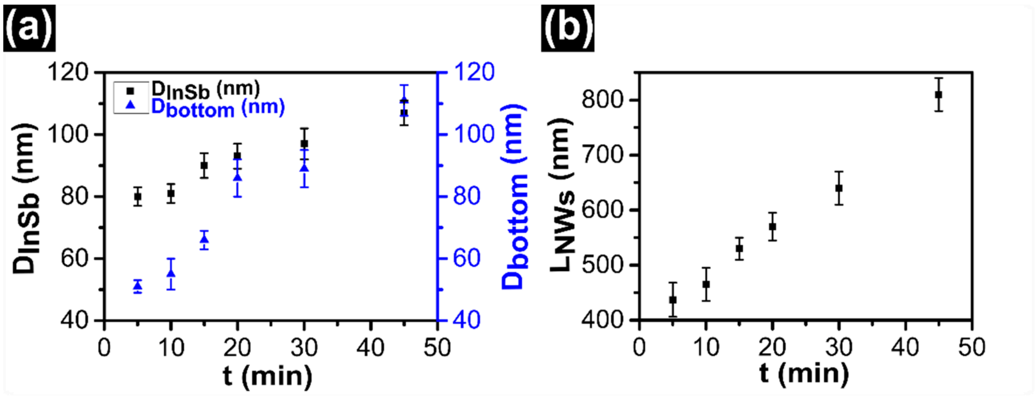

Figure S3. Morphological parameters of InSb/InAs QD NWs, obtained with fixed line pressures F_{In} = 0.30 Torr, F_{As} = 0.30 Torr, at T_{InAs} = 440 °C, while the growth time of top InAs segments varies from 5 min to 45 min. (**a**) Expansion of the NW diameter around the InSb QD and at the bottom of NWs as a function of growth time. (**b**) Evolution of total length of NWs as a function of growth time. The symbols represent an average value of each sample obtained by measuring ~ 25 NWs and error bars represent the standard deviation of the average.
